# 4,4′-Bipyridine–3-nitro­benzoic acid (1/2)

**DOI:** 10.1107/S1600536810003594

**Published:** 2010-02-06

**Authors:** Zhen Zhu, Feng-Qin Wang, Yong-Nan Zhao

**Affiliations:** a College of Materials and Engineering, Tianjin Polytechnic University, Tianjin 300160, People’s Republic of China; b College of Environment and Chemical Engineering & Tianjin Key Laboratory of Fiber Modification and Functional Fiber, Tianjin Polytechnic University, Tianjin 300160, People’s Republic of China

## Abstract

The title compound, C_10_H_8_N_2_·2C_7_H_5_NO_4_,was obtained unintentionally as the harvested product of the hydro­thermal reaction between Co(OAc)_2_·4H_2_O and 4,4′-bipyridine in the presence of 3-nitro­phthalic acid. In the reaction, 3-nitro­phthalic acid is transformed into 3-nitro­benzoic acid by an *in situ* deca­rboxylation reaction, in which the carboxyl­ate group is not deprotonated and is uncoordinated. In the crystal, the uncoordinated 3-nitro­benzoic acid and free 4,4′-bipyridine mol­ecules are linked alternately by O—H⋯N hydrogen bonds into chains, which are assembled by C—H⋯O hydrogen bonds into a three-dimensional supra­molecular network.

## Related literature

For the use of 3-nitro­phthalic acid in the self-assembly of coordination compounds, see: Deng *et al.* (2007*a* ▶,*b* ▶); Huang *et al.* (2007 ▶); Song *et al.* (2007 ▶); Wang *et al.* (2009 ▶).
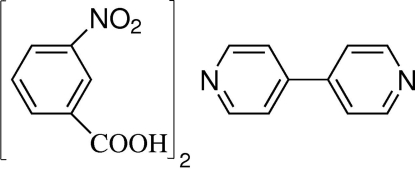


## Experimental

### 

#### Crystal data

C_10_H_8_N_2_·2C_7_H_5_NO_4_
*M* *_r_* = 490.42
Monoclinic, 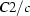

*a* = 26.489 (7) Å
*b* = 6.7757 (14) Å
*c* = 13.291 (3) Å
β = 112.19 (3)°
*V* = 2208.8 (9) Å^3^
*Z* = 4
Mo *K*α radiation
μ = 0.11 mm^−1^
*T* = 113 K
0.20 × 0.12 × 0.10 mm

#### Data collection

Rigaku Saturn CCD area-detector diffractometer
Absorption correction: multi-scan (*CrystalClear*; Rigaku/MSC, 2005 ▶) *T*_min_ = 0.978, *T*_max_ = 0.989
7177 measured reflections
1935 independent reflections
1646 reflections with *I* > 2σ(*I*)
*R*_int_ = 0.029

#### Refinement

*R*[*F* ^2^ > 2σ(*F* ^2^)] = 0.038
*wR*(*F* ^2^) = 0.103
*S* = 1.09
1935 reflections
166 parameters
1 restraint
H atoms treated by a mixture of independent and constrained refinement
Δρ_max_ = 0.18 e Å^−3^
Δρ_min_ = −0.24 e Å^−3^

### 

Data collection: *CrystalClear* (Rigaku/MSC, 2005 ▶); cell refinement: *CrystalClear*; data reduction: *CrystalClear*; program(s) used to solve structure: *SHELXS97* (Sheldrick, 2008 ▶); program(s) used to refine structure: *SHELXL97* (Sheldrick, 2008 ▶); molecular graphics: *SHELXTL* (Sheldrick, 2008 ▶); software used to prepare material for publication: *SHELXTL*.

## Supplementary Material

Crystal structure: contains datablocks I, global. DOI: 10.1107/S1600536810003594/bg2325sup1.cif

Structure factors: contains datablocks I. DOI: 10.1107/S1600536810003594/bg2325Isup2.hkl

Additional supplementary materials:  crystallographic information; 3D view; checkCIF report

## Figures and Tables

**Table 1:** Hydrogen-bond geometry (Å, °)

*D*—H⋯*A*	*D*—H	H⋯*A*	*D*⋯*A*	*D*—H⋯*A*
O2—H2⋯N2^i^	0.85 (1)	1.76 (1)	2.608 (2)	175 (2)
C5—H5⋯O3^ii^	0.93	2.49	3.390 (2)	162
C9—H9⋯O4^iii^	0.93	2.55	3.436 (2)	159
C12—H12⋯O1^iv^	0.93	2.35	3.242 (2)	160

Symmetry codes: (i) 
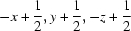
; (ii) 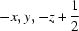; (iii) 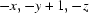; (iv) 
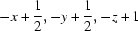
.

**Fig. 1.**
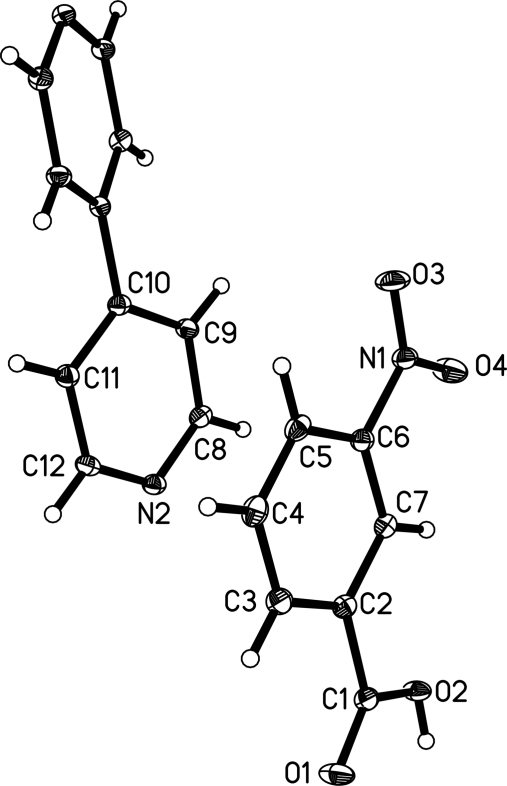
A view of the molecular structure of the title compound, showing the atom-numbering scheme. Dispacement ellipsoids are drawn at the 30% probability level.

**Fig. 2.**
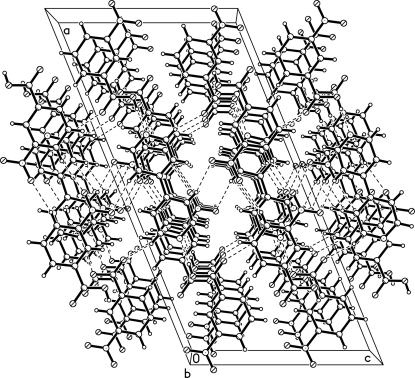
The crystal packing of the title compound along b axis. Hydrogen bonds are indicated by dashed lines.

## supplementary crystallographic information

### Comment

3-nitrophthic acid acting as a multifunctional organic ligand has been widely used in the self-assembly of various coordination compounds (Deng *et al.*, 2007*a*,b; Huang *et al.*, 2007; Song *et al.*, 2007, Wang *et al.*, 2009). The title compound were obtained unintentionally as the harvested product of the hydrothermal reaction between Co(oAc)_2_.4H_2_O and 4,4'-bipyridine in the presence of 3-nitrophthic acid. In the title compound, 3-nitrophthlic acid is transformed into 3-nitrobenzoic acid by *in situ* decarboxylation reaction, in which the carboxylate group is not deprotoned and is uncoordinated. The molecular structure of the title compound is illustrated in Fig. 1. The bond distances and angles are normal within experimental error.

The crystal packing of the title compound is illustrated in Fig. 2. The uncoordinated 3-nitrobenzoic acid and free 4,4'-bipyridine molecules are linked alternately by hydrogen bonds (O—H···O) into one-dimensional chains. Furthermore, these one-dimensional chains are assembled by hydrogen bonds(C—H···O) into three-dimensional supramolecular network.

### Experimental

A mixture of 3-nitrophthalic acid(0.020 g, 0.1 mmol), Co(oAc)_2_.4H_2_O(0.025 g, 0.1 mmol), 4,4'-bipyridine (0.019 g, 0.1 mmol), deionized water (8 ml) was sealed in a Teflon-lined stainless steel vessel (23 ml) and heated at 160 °C for 4 days under autogenous pressure and then cooled slowly to room temperature. The solution was filtered and after allowed to stand for a few weeks at room temperature, purple-red crystals were obtained.

### Refinement

The O-H hydrogen atom was found in a difference Fourier map and fixed during refinement at a O–H distance of 0.85 Å, with *U*_iso_(H)=1.2 *U*_eq_(O). The H atoms of C–H and N–H groups were treated as riding, with C–H = 0.97 Å and N–H = 0.86 Å and *U*_iso_ (H) = 1.2 *U*_eq_(C,*N*).

### Crystal data

**Table:**

C_10_H_8_N_2_·2C_7_H_5_NO_4_	*F*(000) = 1016
*M**_r_* = 490.42	*D*_x_ = 1.475 Mg m^−^^3^
Monoclinic, *C*2/*c*	Mo *K*α radiation, λ = 0.71073 Å
Hall symbol: -C 2yc	Cell parameters from 2874 reflections
*a* = 26.489 (7) Å	θ = 3.1–27.4°
*b* = 6.7757 (14) Å	µ = 0.11 mm^−^^1^
*c* = 13.291 (3) Å	*T* = 113 K
β = 112.19 (3)°	Plate, purple–red
*V* = 2208.8 (9) Å^3^	0.20 × 0.12 × 0.10 mm
*Z* = 4	

### Data collection

**Table:**

Rigaku Saturn CCD area-detector diffractometer	1935 independent reflections
Radiation source: rotating anode	1646 reflections with *I* > 2σ(*I*)
confocal	*R*_int_ = 0.029
Detector resolution: 7.31 pixels mm^-1^	θ_max_ = 25.0°, θ_min_ = 3.1°
ω and φ scans	*h* = −31→31
Absorption correction: multi-scan (*CrystalClear*; Rigaku/MSC, 2005)	*k* = −7→8
*T*_min_ = 0.978, *T*_max_ = 0.989	*l* = −13→15
7177 measured reflections	

### Refinement

**Table:**

Refinement on *F*^2^	Primary atom site location: structure-invariant direct methods
Least-squares matrix: full	Secondary atom site location: difference Fourier map
*R*[*F*^2^ > 2σ(*F*^2^)] = 0.038	Hydrogen site location: inferred from neighbouring sites
*wR*(*F*^2^) = 0.103	H atoms treated by a mixture of independent and constrained refinement
*S* = 1.09	*w* = 1/[σ^2^(*F*_o_^2^) + (0.0674*P*)^2^ + 0.0461*P*] where *P* = (*F*_o_^2^ + 2*F*_c_^2^)/3
1935 reflections	(Δ/σ)_max_ < 0.001
166 parameters	Δρ_max_ = 0.18 e Å^−^^3^
1 restraint	Δρ_min_ = −0.24 e Å^−^^3^

### Special details

**Table:**

Geometry. All e.s.d.'s (except the e.s.d. in the dihedral angle between two l.s. planes) are estimated using the full covariance matrix. The cell e.s.d.'s are taken into account individually in the estimation of e.s.d.'s in distances, angles and torsion angles; correlations between e.s.d.'s in cell parameters are only used when they are defined by crystal symmetry. An approximate (isotropic) treatment of cell e.s.d.'s is used for estimating e.s.d.'s involving l.s. planes.
Refinement. Refinement of *F*^2^ against ALL reflections. The weighted *R*-factor *wR* and goodness of fit *S* are based on *F*^2^, conventional *R*-factors *R* are based on *F*, with *F* set to zero for negative *F*^2^. The threshold expression of *F*^2^ > σ(*F*^2^) is used only for calculating *R*-factors(gt) *etc*. and is not relevant to the choice of reflections for refinement. *R*-factors based on *F*^2^ are statistically about twice as large as those based on *F*, and *R*- factors based on ALL data will be even larger.

### Fractional atomic coordinates and isotropic or equivalent isotropic displacement parameters (Å^2^)

**Table:**

	*x*	*y*	*z*	*U*_iso_*/*U*_eq_	
O1	0.30213 (4)	0.58310 (15)	0.39895 (8)	0.0316 (3)	
O2	0.26214 (4)	0.60692 (13)	0.21797 (8)	0.0238 (3)	
H2	0.2955 (4)	0.608 (2)	0.2257 (14)	0.036*	
O3	0.01600 (4)	0.66151 (15)	0.13387 (9)	0.0328 (3)	
O4	0.06802 (4)	0.65732 (16)	0.04209 (8)	0.0363 (3)	
N1	0.06088 (4)	0.64662 (15)	0.12810 (10)	0.0223 (3)	
N2	0.13751 (4)	0.10542 (14)	0.27135 (9)	0.0189 (3)	
C1	0.26146 (5)	0.59392 (18)	0.31583 (11)	0.0204 (3)	
C2	0.20523 (5)	0.59219 (17)	0.31772 (11)	0.0189 (3)	
C3	0.19834 (5)	0.56014 (18)	0.41546 (11)	0.0231 (3)	
H3	0.2287	0.5420	0.4791	0.028*	
C4	0.14675 (6)	0.55513 (18)	0.41864 (12)	0.0247 (3)	
H4	0.1427	0.5334	0.4843	0.030*	
C5	0.10097 (5)	0.58232 (18)	0.32441 (11)	0.0224 (3)	
H5	0.0661	0.5781	0.3256	0.027*	
C6	0.10882 (5)	0.61589 (17)	0.22863 (11)	0.0188 (3)	
C7	0.15989 (5)	0.62214 (17)	0.22295 (11)	0.0181 (3)	
H7	0.1637	0.6458	0.1573	0.022*	
C8	0.09648 (5)	0.14365 (18)	0.17646 (11)	0.0200 (3)	
H8	0.1049	0.1696	0.1158	0.024*	
C9	0.04234 (5)	0.14626 (18)	0.16471 (11)	0.0193 (3)	
H9	0.0152	0.1758	0.0978	0.023*	
C10	0.02882 (5)	0.10427 (17)	0.25412 (11)	0.0175 (3)	
C11	0.07148 (5)	0.06255 (18)	0.35231 (11)	0.0195 (3)	
H11	0.0642	0.0326	0.4138	0.023*	
C12	0.12465 (5)	0.06609 (17)	0.35760 (11)	0.0197 (3)	
H12	0.1527	0.0400	0.4239	0.024*	

### Atomic displacement parameters (Å^2^)

**Table:**

	*U*^11^	*U*^22^	*U*^33^	*U*^12^	*U*^13^	*U*^23^
O1	0.0164 (5)	0.0538 (7)	0.0208 (6)	−0.0010 (4)	0.0028 (4)	0.0011 (4)
O2	0.0140 (5)	0.0381 (6)	0.0195 (5)	0.0001 (4)	0.0066 (4)	0.0023 (4)
O3	0.0145 (5)	0.0425 (6)	0.0437 (7)	0.0011 (4)	0.0134 (5)	−0.0002 (5)
O4	0.0229 (6)	0.0611 (7)	0.0243 (6)	0.0058 (5)	0.0083 (5)	0.0070 (5)
N1	0.0167 (6)	0.0213 (6)	0.0295 (7)	0.0001 (4)	0.0095 (5)	−0.0005 (5)
N2	0.0153 (6)	0.0180 (6)	0.0226 (6)	0.0008 (4)	0.0064 (5)	−0.0013 (4)
C1	0.0190 (7)	0.0219 (7)	0.0205 (7)	−0.0017 (5)	0.0075 (6)	−0.0003 (5)
C2	0.0185 (7)	0.0183 (6)	0.0203 (7)	−0.0013 (5)	0.0078 (6)	−0.0015 (5)
C3	0.0233 (7)	0.0260 (7)	0.0195 (7)	−0.0018 (5)	0.0076 (6)	0.0001 (5)
C4	0.0302 (8)	0.0256 (7)	0.0235 (8)	−0.0019 (6)	0.0161 (6)	0.0002 (6)
C5	0.0227 (8)	0.0179 (6)	0.0321 (8)	−0.0014 (5)	0.0167 (6)	−0.0026 (5)
C6	0.0169 (7)	0.0154 (6)	0.0233 (8)	−0.0005 (5)	0.0067 (6)	−0.0018 (5)
C7	0.0201 (7)	0.0166 (6)	0.0198 (7)	−0.0013 (5)	0.0100 (6)	−0.0018 (5)
C8	0.0189 (7)	0.0192 (7)	0.0233 (8)	−0.0017 (5)	0.0096 (6)	−0.0009 (5)
C9	0.0150 (7)	0.0197 (7)	0.0209 (7)	0.0005 (5)	0.0042 (6)	0.0006 (5)
C10	0.0153 (7)	0.0147 (6)	0.0220 (7)	−0.0007 (5)	0.0067 (6)	−0.0025 (5)
C11	0.0187 (7)	0.0200 (6)	0.0203 (7)	0.0009 (5)	0.0079 (6)	0.0001 (5)
C12	0.0150 (7)	0.0197 (7)	0.0211 (7)	0.0018 (5)	0.0030 (6)	−0.0009 (5)

### Geometric parameters (Å, °)

**Table:**

O1—C1	1.2197 (17)	C4—H4	0.9300
O2—C1	1.3106 (17)	C5—C6	1.3839 (19)
O2—H2	0.850 (9)	C5—H5	0.9300
O3—N1	1.2242 (14)	C6—C7	1.3838 (18)
O4—N1	1.2294 (14)	C7—H7	0.9300
N1—C6	1.4695 (18)	C8—C9	1.3827 (18)
N2—C12	1.3402 (18)	C8—H8	0.9300
N2—C8	1.3430 (18)	C9—C10	1.3938 (19)
C1—C2	1.4991 (19)	C9—H9	0.9300
C2—C7	1.388 (2)	C10—C11	1.3950 (19)
C2—C3	1.3954 (19)	C10—C10^i^	1.489 (2)
C3—C4	1.3835 (19)	C11—C12	1.3836 (18)
C3—H3	0.9300	C11—H11	0.9300
C4—C5	1.388 (2)	C12—H12	0.9300
			
C1—O2—H2	106.5 (12)	C7—C6—N1	118.22 (12)
O3—N1—O4	123.21 (12)	C5—C6—N1	118.77 (11)
O3—N1—C6	118.74 (12)	C6—C7—C2	118.33 (13)
O4—N1—C6	118.05 (10)	C6—C7—H7	120.8
C12—N2—C8	117.68 (11)	C2—C7—H7	120.8
O1—C1—O2	124.35 (13)	N2—C8—C9	123.06 (13)
O1—C1—C2	121.85 (13)	N2—C8—H8	118.5
O2—C1—C2	113.79 (12)	C9—C8—H8	118.5
C7—C2—C3	119.66 (13)	C8—C9—C10	119.38 (12)
C7—C2—C1	120.54 (12)	C8—C9—H9	120.3
C3—C2—C1	119.80 (12)	C10—C9—H9	120.3
C4—C3—C2	120.68 (13)	C9—C10—C11	117.44 (12)
C4—C3—H3	119.7	C9—C10—C10^i^	121.59 (14)
C2—C3—H3	119.7	C11—C10—C10^i^	120.97 (15)
C3—C4—C5	120.38 (13)	C12—C11—C10	119.56 (13)
C3—C4—H4	119.8	C12—C11—H11	120.2
C5—C4—H4	119.8	C10—C11—H11	120.2
C6—C5—C4	117.93 (12)	N2—C12—C11	122.87 (12)
C6—C5—H5	121.0	N2—C12—H12	118.6
C4—C5—H5	121.0	C11—C12—H12	118.6
C7—C6—C5	123.01 (13)		
			
O1—C1—C2—C7	174.30 (12)	O4—N1—C6—C5	−173.13 (11)
O2—C1—C2—C7	−6.02 (16)	C5—C6—C7—C2	0.37 (18)
O1—C1—C2—C3	−5.73 (18)	N1—C6—C7—C2	−179.69 (10)
O2—C1—C2—C3	173.94 (10)	C3—C2—C7—C6	−1.03 (17)
C7—C2—C3—C4	0.92 (18)	C1—C2—C7—C6	178.94 (10)
C1—C2—C3—C4	−179.05 (11)	C12—N2—C8—C9	0.77 (17)
C2—C3—C4—C5	−0.11 (18)	N2—C8—C9—C10	−1.16 (18)
C3—C4—C5—C6	−0.54 (18)	C8—C9—C10—C11	0.43 (16)
C4—C5—C6—C7	0.42 (18)	C8—C9—C10—C10^i^	−179.76 (8)
C4—C5—C6—N1	−179.52 (10)	C9—C10—C11—C12	0.59 (17)
O3—N1—C6—C7	−172.63 (10)	C10^i^—C10—C11—C12	−179.22 (8)
O4—N1—C6—C7	6.93 (16)	C8—N2—C12—C11	0.33 (17)
O3—N1—C6—C5	7.31 (16)	C10—C11—C12—N2	−1.01 (18)

Symmetry codes: (i) −*x*, *y*, −*z*+1/2.

### Hydrogen-bond geometry (Å, °)

**Table:**

*D*—H···*A*	*D*—H	H···*A*	*D*···*A*	*D*—H···*A*
O2—H2···N2^ii^	0.85 (1)	1.76 (1)	2.608 (2)	175 (2)
C5—H5···O3^i^	0.93	2.49	3.390 (2)	162
C9—H9···O4^iii^	0.93	2.55	3.436 (2)	159
C12—H12···O1^iv^	0.93	2.35	3.242 (2)	160

Symmetry codes: (ii) −*x*+1/2, *y*+1/2, −*z*+1/2; (i) −*x*, *y*, −*z*+1/2; (iii) −*x*, −*y*+1, −*z*; (iv) −*x*+1/2, −*y*+1/2, −*z*+1.

## References

[bb1] Deng, Y. H., Liu, J., Yang, Y. L., Zhu, H. J. & Ma, H. W. (2007*a*). *Chin. J. Struct. Chem.* **26**, 642–648.

[bb2] Deng, Y. H., Wang, S. Y., Liu, J., Yang, Y. L., Zhang, F. & Ma, H. W. (2007*b*). *Acta Chim. Sin.* **65**, 809–815.

[bb3] Huang, Y., Yan, B., Shao, M. & Chen, Z. X. (2007). *J. Mol. Struct.* **871**, 59–66.

[bb4] Rigaku/MSC (2005). *CrystalClear* Rigaku/MSC, The Woodlands, Texas, USA.

[bb5] Sheldrick, G. M. (2008). *Acta Cryst.* A**64**, 112–122. 10.1107/S0108767307043930 18156677

[bb6] Song, Y. S., Yan, B. & Chen, Z. X. (2007). *Appl. Organomet. Chem.* **21**, 150–155.

[bb7] Wang, F.-Q., Lu, F.-L., Wei, B. & Zhao, Y.-N. (2009). *Acta Cryst.* C**65**, m42–m44. 10.1107/S0108270108041504 19129598

